# Perivascular epithelioid cell tumor of the descending colon mimicking a gastrointestinal stromal tumor: a case report

**DOI:** 10.1186/s12957-016-1046-7

**Published:** 2016-11-14

**Authors:** Ryuta Iwamoto, Tatsuki R. Kataoka, Ayako Furuhata, Kazuo Ono, Seiichi Hirota, Kenji Kawada, Yoshiharu Sakai, Hironori Haga

**Affiliations:** 1 Department of Diagnostic Pathology, Kyoto University Hospital, 54 Syogoinkawahara-cho, Sakyo-ku, Kyoto, 606-8507 Japan; 2 Department of Pathology, Japan Red Cross Society Wakayama Medical Center, 4-20 Komatsubara-dori, Wakayama, 640-8558 Japan; 3 Department of Surgical Pathology, Hyogo College of Medicine, 1-1 Mukogawa-cho, Nishinomiya, Hyogo 663-8501 Japan; 4 Department of Surgery, Kyoto University Hospital, 54 Syogoinkawahara-cho, Sakyo-ku, Kyoto, 606-8507 Japan

**Keywords:** Gastrointestinal stromal tumor, KIT, Perivascular epithelioid cell tumor, Platelet-derived growth factor receptor

## Abstract

**Background:**

We present a case of perivascular epithelioid cell tumor (PEComa), which clinically and histologically mimics a gastrointestinal stromal tumor (GIST).

**Case presentation:**

A 42-year-old woman was found to have a mass in the left flank during her annual medical checkup. Computed tomography examination revealed a submucosal tumor of the descending colon. Surgeons and radiologists suspected that the lesion was a GIST, and left hemicolectomy was performed without biopsy. Microscopic examination showed that the lesion was composed of spindle and epithelioid cells, which were immunohistochemically negative for c-kit and positive for platelet-derived growth factor receptor (PDGFR) α. Initial diagnosis of PDGFRα-positive GIST was made. However, gene analysis did not reveal mutations in PDGFRα. Additional immunohistochemistry showed that tumor cells were positive for human melanin black 45 (HMB45), melanA, and the myogenic marker calponin. A final diagnosis of PEComa was made.

**Conclusion:**

PEComa should be included in the differential diagnosis of PDGFRα-positive spindle cell tumors in the wall of the gastrointestinal tract.

## Background

Gastrointestinal stromal tumor (GIST) is the most common mesenchymal tumor in the walls of the gastrointestinal tract [1]. GISTs typically harbor gain-of-function type mutations in the KIT genes [2], and GISTs without KIT mutations have gain-of-function type mutations in the platelet-derived growth factor receptor (PDGFR) α genes [3]. Expression of the two genes is mutually exclusive [1–3]. Perivascular epithelioid cell tumor (PEComa) is a less common mesenchymal tumor, expressing melanocytic and myogenic markers such as actin, desmin, calponin, human melanin black (HMB) 45, melanA, and microphthalmia-associated transcription factor (MITF) [4]. PEComa can occur in any organs, but is rarely detected in the gastrointestinal wall [5]. Herein, we report a case of PDGFRα-positive PEComa arising in the wall of the descending colon.

## Case presentation

**Fig. 1:**
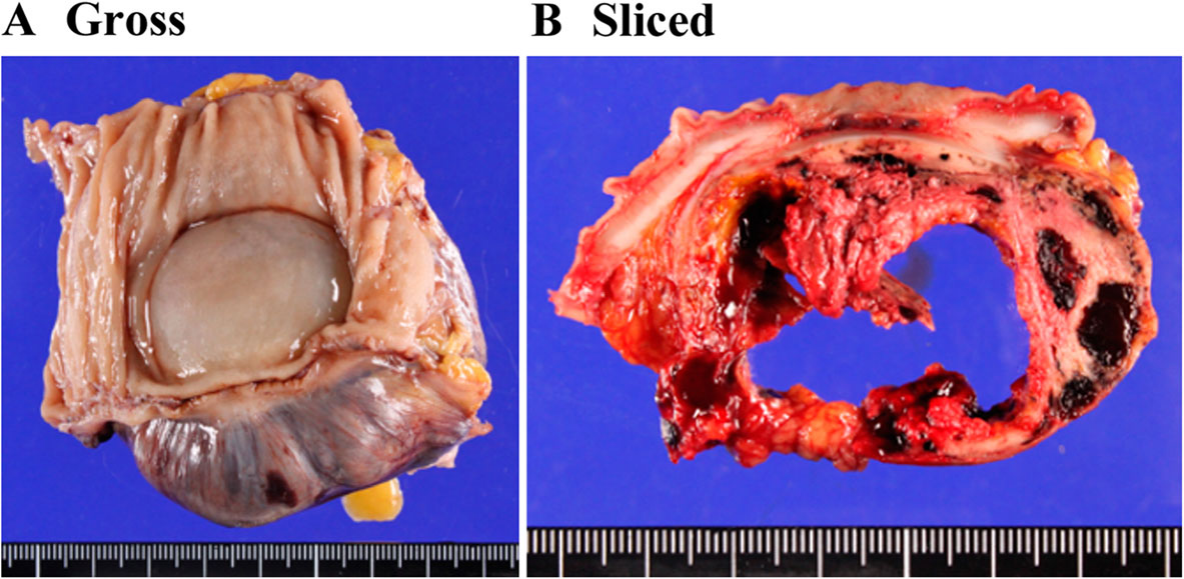
Macroscopic findings. **a** Gross appearance. **b** Sliced specimens

**Fig. 2:**
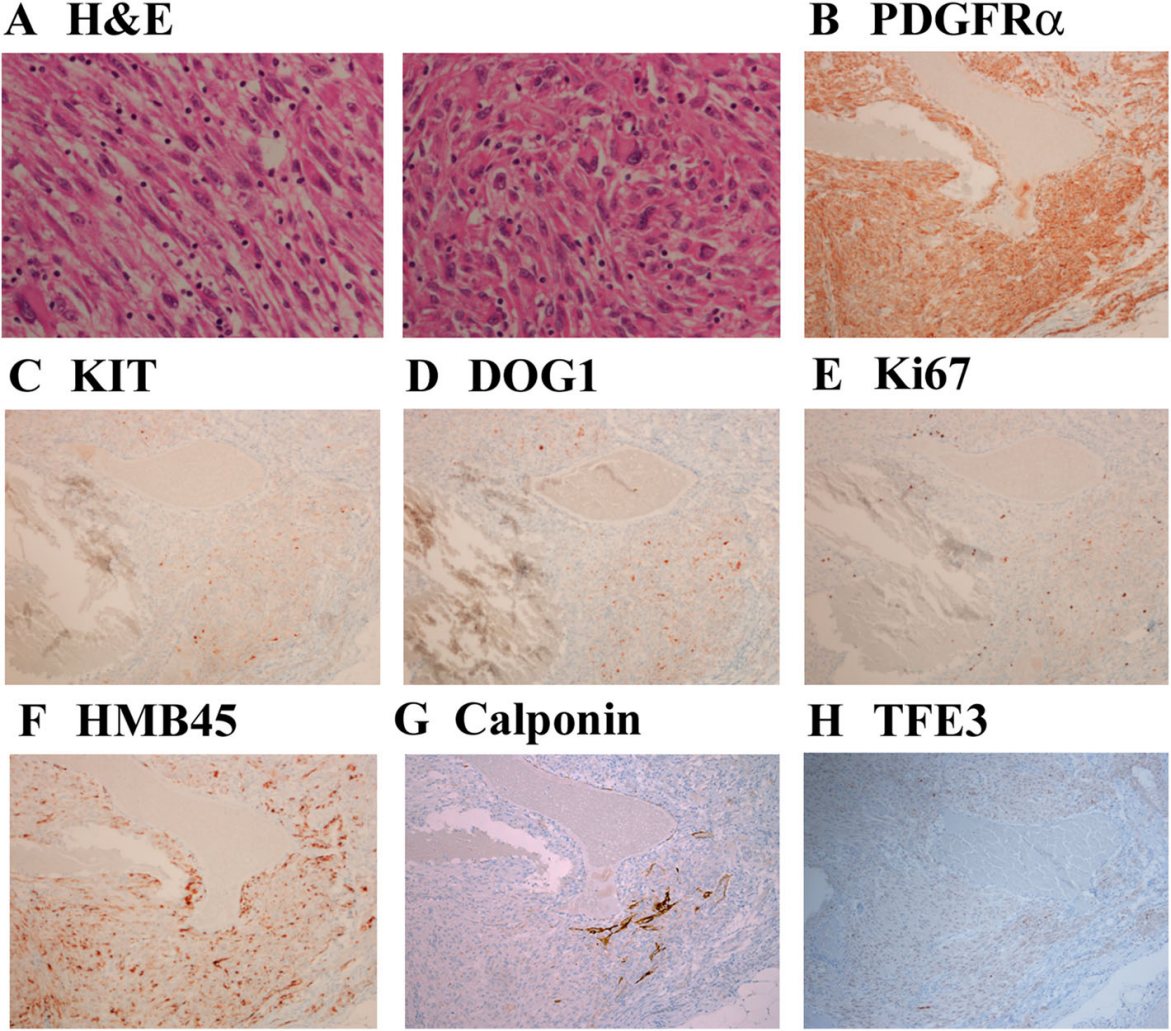
Histological findings. **a** Hematoxylin and eosin (H&E) staining. Two representative fields. Immunohistochemical specimens for **b** PDGFRα, **c** KIT, **d** discovered on GIST-1 (DOG1), **e** Ki67, **f** HMB45, **g** Calponin, and **h** TFE3. Photos are ×200 magnification in **a** and ×100 magnification in **b**–**h**

A 42-year-old woman underwent abdominal ultrasonography during her annual medical checkup and a mass in her left flank region was identified. She was admitted to the hospital for further examination. A computed tomography scan and endoscopic examination revealed a submucosal tumor in the wall of the descending colon. Systemic magnetic resonance imaging and positron emission tomography scans did not show any other lesions. The lesion was suspected to be a colonic GIST and left hemicolectomy was performed. Upon macroscopic examination, the tumor was 5 cm in the greatest dimension, well-circumscribed but uncapsulated, and extended from the muscular propria into the subserosa (Fig. 1a). The cut surface was hemorrhagic and necrotic (Fig. 1b). Microscopically, the tumor cells consisted of spindle and epithelioid cells with a granular cytoplasm (Fig. 2a). Based on the clinical diagnosis of GIST, a panel of immunohistochemistry including KIT, PDGFRα, discovered on GIST-1 (DOG1), CD34, S100, desmin, and Ki67 were performed. The tumor cells were positive for PDGFRα (Fig. 2b) and negative for KIT (Fig. 2c), DOG1 (Fig. 2d), CD34, S100, and desmin. The Ki-67 index was 3% (Fig. 2e). We initially suspected the tumor to be a PDGFRα-positive GIST. Mutational analysis did not reveal any mutation in PDGFRα or KIT, and suggested the possibility of a low-grade tumor other than GIST. Upon further examination, the tumor cells were found to be positive for HMB45 (Fig. 2f) and calponin (Fig. 2g), and negative for melanA, MITF, SOX10, and actin. These results were compatible with PEComa. This tumor was immunohistochemically negative for TFE3 (Fig. 2h), but did not show rearrangement of TFE3 in fluorescence in situ hybridization (FISH) (data not shown). The patient was alive without recurrence 5 months after the resection.

## Discussion

PEComa is rare in the gastrointestinal tract. To the best of our knowledge, only 36 cases of gastrointestinal PEComa have been reported sporadically [6, 7]. Doyle et al. performed a clinicopathologic study of 35 cases of gastrointestinal PEComa [5]. The current case shows similarities with previously reported cases of gastrointestinal PEComa, in terms of the clinicopathological features and immunological profile. GIST does not show immunoreactivity for melanocytic markers [8], and expression of HMB45 is important to support the diagnosis of PEComa. Metastatic melanoma is positive for HMB45, but is also positive for S100 protein and lacks expression of myogenic markers such as calponin. Some cases of PEComa show gene rearrangement involving TFE3, and strong nuclear TFE3 expression [4, 5]. In our case, TFE3 rearrangement was not detected by FISH. This result is not incompatible with a diagnosis of PEComa because most gastrointestinal PEComas are negative for TFE3 [5]. Thus, TFE3 status may not be a diagnostic clue in gastrointestinal PEComa.

The tumor cells in our case were partly epithelioid and immunohistochemically PDGFRα-positive. These phenotypes were thought to be compatible with the initial diagnosis of GISTs with a PDGFRα mutation [3, 9]. However, GISTs with a PDGFRα mutation most commonly arise in the stomach [9, 10], and the tumor is typically DOG1-positive [11, 12]. In contrast to the case of GISTs, PDGFRα positivity and mutations in PDGFRα genes have not been reported in PEComas, to the best of our knowledge. The status of PDGFRα in PEComa should be further studied to diagnose PDGFRα-positive mesenchymal tumors in the gastrointestinal tract. Both GISTs and PEComas are treated with surgical resection and chemotherapy. GISTs are susceptible to the tyrosine kinase inhibitor imatinib [10], although PEComas are susceptible to another inhibitor, sirolimus [13]. Therefore, distinguishing between GISTs and PEComas would be important for appropriate administration of kinase inhibitors.

## Conclusion

PEComa should be included in the differential diagnosis of mesenchymal tumors in the wall of the gastrointestine, even though tumor cells are immunohistochemically PDGFRα-positive. Mutational analysis should be performed to confirm the diagnosis of GIST, even though PDGFRα is immunohistochemically positive.

## Abbreviations

DOG1: Discovered on GIST-1
GIST: Gastrointestinal stromal tumor
HMB45: Human melanin black 45
MITF: Microphthalmia-associated transcription factor
PDGFR: Platelet-derived growth factor receptor
PEComa: Perivascular epithelioid cell tumor
TFE3: Transcriptional factor E3
